# Predicting potential recovery of the endangered bromeliad *Tillandsia utriculata*: An agent-based modeling approach

**DOI:** 10.1371/journal.pcbi.1013157

**Published:** 2025-06-10

**Authors:** Alexandra M. Campbell, Anna C. Kula, Rachel S. Jabaily, Brad Oberle, Brian Sidoti, Alex Capaldi, Erin N. Bodine

**Affiliations:** 1 Department of Mathematics and Statistics, Rhodes College, Memphis, Tennessee, United States of America; 2 Department of Organismal Biology and Ecology, Colorado College, Colorado Springs, Colorado, United States of America; 3 New York Botanical Gardens, Bronx, New York, United States of America; 4 Marie Selby Botanical Garden, Sarasota, Florida, United States of America; 5 The Kampong, National Tropical Botanical Garden, Miami, Florida, United States of America; 6 Department of Mathematics and Statistics, James Madison University, Harrisonburg, Virginia, United States of America; 7 Department of Mathematics and Statistics, Valparaiso University, Valparaiso, Indiana, United States of America; The University of Melbourne, AUSTRALIA

## Abstract

Invasive pests and pathogens are a major driver of biodiversity loss. Some rare species may persist through rapid evolution to tolerate or escape new threats, but representing the underlying ecological and evolutionary processes at the appropriate scale is analytically and computationally challenging. *Tillandsia utriculata* has been classified as endangered in Florida where its population has decreased significantly due to predation by the invasive Mexican weevil *Metamasius callizona*. Adult female weevils deposit their eggs in leaves of epiphytic bromeliads, preferentially ovipositing in the largest rosettes. Once the eggs hatch, the larvae consume the core of the rosette, often leading to pre-reproductive death. During the past three decades of predation, the *T. utriculata* population has shifted to initiating the production of inflorescences (to commence its single attempt at sexual reproduction) at smaller rosette sizes. Importantly, the rosette size at induction is correlated with the number of seeds produced. We have constructed an agent-based model to simulate the dynamics of a Florida *T. utriculata* population over many generations where the minimum rosettes size required to initiate inflorescence production (minimum size of induction or MSI), is an inherited trait. We use the model to explore how predation may have shifted the population’s genetic composition and the impact this has on population viability. Our results show that larger germination rates are required for population viability when weevils are present. Parameter uncertainty analysis revealed that in the presence of weevil predation, only a population with a very high germination rate and a short period of predation would sustain its population for 100 years with sizes similar to simulations without weevil predation. Furthermore, uncertainty analysis showed that the mean MSI of the population decreased over a 100-year period without weevil predation, and this trend was exacerbated by the presence of weevil predation.

## Author summary

The giant air plant of Florida has been declared endangered due to predation from an invasive weevil. These plants are incredibly important to the biodiversity of Florida; they have a water retaining tank within which live ecosystems of thousands of individuals, some that are completely reliant on these types of micro ecosystems. The giant air plants have only a single reproduction event during their lifespans, and the predatory weevils target the reproductive portion of the plants. This has led to the population reproducing earlier, when they are of a smaller size. Our computer simulations have shown that, even in the absence of the weevil, the plants continue to follow this earlier reproduction trend, putting their long-term survival at risk.

## Introduction

Invasive pests pose a globally significant threat to biological diversity [1]. Some of the most dramatic cases involve threatened keystone and foundational species. From plague-stricken prairie dogs to blighted American chestnuts, introduced predators and pathogens have driven once-dominant species towards extinction, with cascading effects for other dependent organisms and ecosystem services [2]. While invasive pests are generally detrimental for biodiversity, conservation outcomes vary with complex interactions between ecological and evolutionary forces across scales. In the case of prairie dogs, colony extinction depends on spatial configuration [3], while genotype influences individual survival and the potential for rapid evolution of resistance [4]. More generally, predators that target adults often drive rapid evolution of faster maturation and smaller body size [5]. Rapid evolution can also promote dispersal through fragmented landscapes [6] and occupation of predation refugia [7]. However, experimental evidence for rapid evolution concentrates on animals and organisms with short generation times. Protecting long-lived keystone species, especially plants, from invasive pests requires simulating where, when and how key ecological and evolutionary forces interact. One example of a long-lived keystone plant that may have experienced rapid evolution due to an invasive pest are *Tillandsia utriculata* populations in Florida, USA.

**Fig 1:**
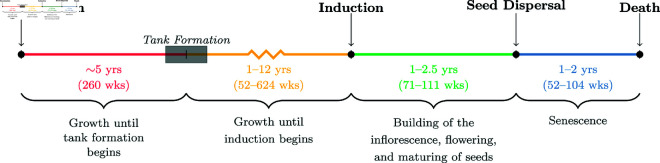
Lifespan of a single *T. utriculata* rosette. The key moments in the lifespan of a *T. utriculata* rosette include germination, tank formation, induction, seed dispersal, and death. The years between these developmental milestones correspond to distinct growth, reproductive, and senescence periods.

*Tillandsia utriculata* L. (Bromeliaceae) is a large, long-lived, epiphytic bromeliad native to Florida, the broader Caribbean, and Central America. Like most bromeliads, the vegetative body of *T. utriculata* consists of leaves arranged in a spiral rosette pattern with a single bud region, called the apical meristem, that controls the production of new tissue [8]. When the rosette is young, the apical meristem produces new leaves. Eventually, the apical meristem converts in a process known as *inflorescence induction* to produce a single, terminal cluster of flowers (an inflorescence) at the top of the rosette that is used in sexual reproduction; Fig 1 shows a timeline of the *T. utriculata* lifespan. Some populations of *T. utriculata* are also capable of reproducing asexually via axillary meristems that produce clonal offshoots called pups. The Florida populations, however, are incapable of producing clonal pup rosettes and rely exclusively on sexual reproduction through the apical meristem[9]. Since a *T. utriculata* rosette produces only one inflorescence in its lifetime, this means that in Florida, each *T. utriculata* rosette can have only one reproductive event in its lifetime (known as a semelparous life history strategy).

Since the late 1980s, large bromeliads in Florida (including *T. utriculata*) have been subject to predation from the invasive weevil *Metamasius callizona* [10,11]. Adult *M. callizona* feed on the leaves of bromeliads, and though this can be harmful to the plants, it is not necessarily lethal. Lethal damage to bromeliad rosettes is caused by *M. callizona* in the larval stage. Adult female weevils lay their eggs in slits they cut into the base of large rosettes. Once the eggs hatch, the larvae bore into and feed upon the available tissue, including meristematic tissue. For those species capable of producing clonal pup rosettes (including the large, Florida native epiphytic bromeliads *Tillandsia fasciculata* and *Guzmania monostachia*, which may maintain their ability to generate pups after weevil infestation), there is a possibility of future reproduction through axillary meristem regrowth. However, *T. utriculata* populations in Florida rely exclusively on the production of an inflorescence for reproduction, and thus there is no possibility of future reproduction once weevil predation destroys the apical meristem. While all the large Florida bromeliads (*T. utriculata*, *T. fasciculata*, and *G. monostachia*) have been heavily impacted by the weevil and are currently listed as endangered in the state of Florida [12], *T. utriculata* is currently of greater conservation concern due to its semelparous life history strategy [10,11].

The ecology of *M. callizona* and native Florida bromeliad populations have been of considerable interest to researchers, conservationists, and bromeliad horticulturists for the past two decades. In the past decade, there has been some evidence that the bromeliads in Myakka River State Park (MRSP) in Sarasota County, FL are initiating induction of the inflorescence at smaller sizes than has been seen in the past [field observations by T. Cooper]. One possible explanation for this shift is that *M. callizona*, which preferentially select larger rosettes for ovipositing eggs, have exerted sufficient pressure on the bromeliad populations to select for earlier timing of induction [13]. With seed fecundity correlated to longest leaf length (LLL) at time of inflorescence production [14], a shift to induction occurring at smaller rosette sizes would correlate to a lower mean seed fecundity within the population. A significant shift in average seed fecundity along with high seed mortality and low germination rate would likely correlate to lowered population viability. To explore this possibility, we have constructed an agent-based model (ABM) to simulate the population dynamics of *T. utriculata* in an area of MRSP over several generations where the minimum size at which an individual rosette can initiate inflorescence production, called minimum size of induction (MSI), is an inherited trait. The protocols within the model are largely structured around the size classes defined by Cooper in [11,15]; see Table 1. Of the three size classes, only the largest two are explicitly modeled (medium and large size classes in Table 1; post tank formation in Fig 1). Not only does this reduce the computational demands of the model to reasonable levels, but it also shifts the focus of the model to the more relevant individuals. In particular, the focus lies on those rosettes that are both subject to weevil predation and capable of reproduction. The small size class (LLL<15 cm) is not a likely location for *M. callizona* oviposition [13], and thus was not explicitly included in the model.

**Table 1:** *Tillandsia utriculata* death rate data by size class.

Size Class	Age (yrs)	LLL (cm)	Death Rate (per wk) due to			
			Senescence	Natural Event	Predation	Other
Small	0 – 5	0 – 15	—	0.01502	0.00719	0.00817
Medium	5 – 12	15 – 50	0.00483	0.00976	0.00708	0.00671
Large	>12	>50	0.00395	0.00400	0.00631	0.00300

Note that only rosettes with LLL ≥ 15 cm are explicitly represented in the model. Size class descriptions and death rate data from [11,15]. The different types of death are senescence (post seed dispersal), natural event (natural death caused when rosette is knocked to the ground in a natural weather event), predation (caused by weevil damage), and other (disappearance, overcrowding, etc).

While there have been several illuminating studies of *T. utriculata* [11,13,16] an unfortunate dearth of quantified information exists for some key demographic parameters, specifically germination rate and mean size at induction. Therefore, with this study, we utilize ABM simulation experiments to determine likely ranges for these parameters. Through simulation experiments we determine the minimum germination rate required to sustain a *T. utriculata* population over 100 years in the presence and absence of *M. callizona*. Additionally, we perform a multidimensional parameter uncertainty analysis that explores the impact of key parameters on population size and population mean MSI over time both with and without the presence of *M. callizona*.

## Results

### Simulation experiment protocols

We created an agent-based model (ABM) to simulate the population dynamics of the *T. utriculata* over many generations. The model includes growth and dispersal procedures informed by its life history and collected data. In particular, the model simulates the MSI as an inherited trait. The model also includes predation by the invasive weevil *M. callizona*, which can be set to occur for different lengths of time. Analysis of this model is intended to explain the impact weevil predation may have had on the timing of reproduction in *T. utriculata*. A complete description of the ABM is given in the Methods and Models section.

The duration of a single simulation was set to 5,200 time steps (1 time step = 1 week), the equivalent of 100 years. We define a single *experiment* as a set of 100 simulations with the same parameter values. Since each simulation is stochastic, we summarize results across a single experiment (100 simulations) to draw conclusions about general model behavior and emergent properties for the given parameter set and forest structure. For each simulation, we recorded the following model output data:

1. The mean MSI over the population of *T. utriculata* with LLL ≥15 cm at every time step (i.e. each week). We define $\mu_{t}$ as the mean MSI at week *t*.
2. The population size of *T. utriculata* with LLL ≥15 cm at the final time step ($N_{\text{f}}$, i.e. 5,200 wks or sooner if the population went extinct prior to 5,200 wks). We define *N*_*t*_ as the total population size at week *t*.

Using model output #2, we calculated the extinction probability for each experiment (i.e. over 100 simulations),

$$P=\frac{\#\text{ simulations with }N_{\text{f}}=0}{100\text{ simulations}},$$ (1)

the median change in population size for each experiment,

$$\Delta N=\text{Median}[N_{\text{f}}-N_{0}].$$ (2)

and using model output #1, we calculated the mean change in the mean MSI for each experiment,

$$\Delta \mu =\text{Mean}[\mu_{\text{f}}-\mu_{0}].$$ (3)

Parameters that were varied between experiments include initial mean MSI ($\mu_{0}$), germination rate (*g*), variation in heritability (ν), standard deviation in initial MSI distribution ($\sigma_{0}$), and years of predation (*T*_*p*_).

### Exploration of the $\left(\mu_{0},g\right)$ parameter space

**Fig 2:**
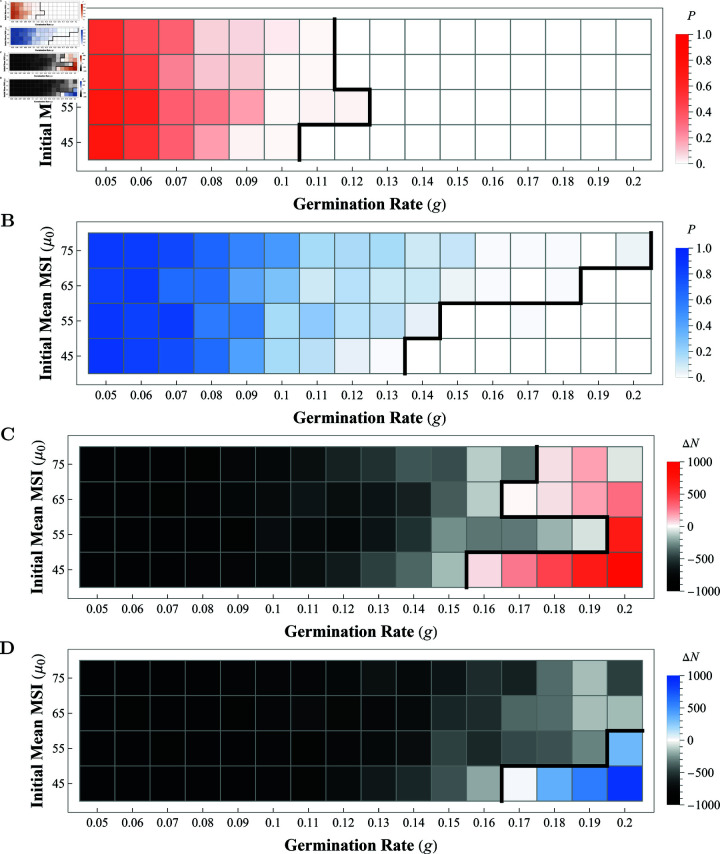
Each square gives extinction probability p (AB) or median population change d (CD) for 100 simulations at $\left(g,\mu_{0}\right)$. Red is without weevil predation (**AC**); blue is with weevil predation (**BD**). Black represents a population decrease over 100 years (**CD**). The thick, black line represents the transition from *p*>0 to *p* = 0 (**AB**) or where *d* = 0 (**CD**).

To determine germination rates which would allow for the Florida *T. utriculata* population to survive over 100 years (i.e., 5,200 weeks), we ran experiments for 64 combinations of initial mean MSI and germination rates with $\mu_{0}\in \{45,55,65,75\}$ and g∈{0.05,0.06,…,0.19,0.20}. For each mean MSI, we determined the minimum germination rate with a zero extinction probability (i.e. *p* = 0), and the minimum germination rate with median population increase by year 100 (i.e. *d*>0). The matrix plots in Fig 2 show the values of *p* and *d* for various combinations of germination rates (*g*, horizontal axis) and initial mean MSI ($\mu_{0}$, vertical axis). The color of each square in the matrix plot shows the value of *p* (Fig 2A and 2B) or *d* (Fig 2C and 2D) as calculated for a single experiment (i.e. 100 simulations). The values of *p* vary from 0 to 1, with 1 representing extinction in every simulation by 5,200 weeks. The values of *d* vary from -1,000 to 1,000, with grey colors representing negative values of *d* (i.e. median population decline at 5,200 weeks), and red (no weevil predation) or blue (with weevil predation) representing positive values of *d* (i.e. median population increase at 5,200 weeks).

Germination rates greater than 10–12% are necessary to avoid extinction without weevil predation and there is little variation over the initial mean MSI value (see Fig 2A). However, germination rates greater than 13–20% are required when weevil predation is present with the minimum required germination rate increasing with initial mean MSI (see Fig 2B). The correlation between extinction probability and germination rate is stronger than between initial mean MSI and extinction probability (over the ranges explored), indicating that the germination rate is more important to the survival of the bromeliads than MSI. Germination rates around 20% are necessary to maintain population size and allow for population growth over 100 years without weevil predation (see Fig 2C). Growth is further inhibited when weevil predation occurs (see Fig 2D).

### Exploration of the $\left(\mu_{0},\sigma_{0},g,\nu ,T_{p}\right)$ parameter space

To determine which parameters had the most influence over the final population size ($N_{\text{f}}$) and the change in the mean MSI at the final time (Δμ) we conducted simulations on a Latin Hypercube Sampling (LHS) of the parameter space. For an LHS of *K* parameters, the parameter space is adequately covered if the number of samples (i.e. number of parameters sets), denoted 𝒩, satisfies the inequality $𝒩>\frac{4}{3}K$ [17]. Our analysis consisted of five varied parameters: initial mean MSI ($\mu_{0}$), initial standard deviation in MSI distribution ($\sigma_{0}$), germination rate (*g*), MSI heritability variation (ν), and the length of the predation period (*T*_*p*_). We additionally calculated partial rank correlation coefficients (PRCCs) to determine to which parameters the final population size and final change in mean MSI were most sensitive. PRCC values range from -1 to 1 with positive values indicating that small increases in the parameter correlate to increases in the measured model outcome and negative PRCCs indicating that small increases in the parameter correlate to decreases in the model outcome; PRCCs whose absolute value is close to 1 have a strong correlation. We calculated the significance of each PRCC with a Student’s *t*-test [17].

**Fig 3:**
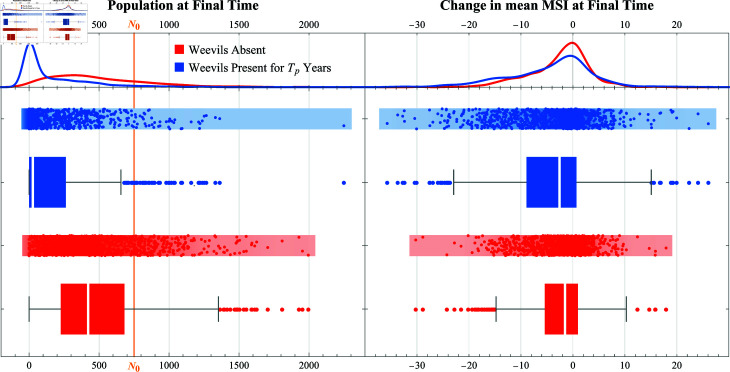
Distribution of population at final time (left) and change in mean MSI at final time (right) from simulations across 1000 parameter sets when weevils are absent (red) and with weevils present for *T*_*p*_ years (blue).

**Analysis with 𝒩=1000 parameter sets.** We generated 𝒩=1000 parameter sets using LHS and ran one simulation for each parameter set. All 1000 simulations were simulated using the same initialized forest (see the Initialization section) and each simulation started with a population of 750 *T. utriculata* rosettes with an initial LLL ≥ 15 cm (i.e., $\ell_{i}(0)\geq 15$ cm). Fig 3 shows the distribution of the population size at the final time (left) and the change in the mean MSI at the final time (right) when the weevils are absent (shown in red) and when the weevils are present for *T*_*p*_ years (shown in blue). In the simulations with no weevils present, only 0.7% of the parameter sets resulted in the *T. utriculata* population going extinct prior to 5200 time steps (i.e., 100 years). In contrast, 44.1% of the simulations with weevils are present for *T*_*p*_ years resulted in the population going extinct before 100 years. Across the 1000 parameter sets, 886 resulted in a lower final population size with weevils present than without, while 589 of the 1000 parameter sets resulted in a lower mean MSI at the final time with weevils present than without. The difference between the median value of the mean MSI at the final time with and without weevil present was significantly different (Mann-Whitney, $p=1.30\;\times \;10^{-6}$). Table 2 shows the PRCC values and corresponding *p*-values for each combination of parameter ($\mu_{0}$, $\sigma_{0}$, *g*, ν, and *T*_*p*_) and outcome ($\text{median}[N_{\text{f}}]$ without and with weevils present, and $\text{mean}[\mu_{5200}-\mu_{0}]$ without and with weevils present). PRCC values indicating a strong correlation (i.e., |PRCC|>0.7) with a low *p* value (i.e., *p*<0.1) are indicated with gray shading in Table 2. Though many of the PRCC values had a *p*-value that met the 90% confidence threshold, none of the PRCC values indicated a strong correlation between a parameter and one of the two measured model outputs. However, this analysis only executed a single simulation for each parameter set.

**Table 2:** PRCC values (with *p*-values in parentheses) for the median final population size and difference in mean MSI at the final time, without weevil predation and with weevil predation.

Parameter	$N_{\text{f}}$ for 𝒩=1000				$\mu_{\text{f}}-\mu_{0}$ for 𝒩=1000			
	without weevil		with weevil		without weevil		with weevil	
	PRCC	*p*-value	PRCC	*p*-value	PRCC	*p*-value	PRCC	*p*-value
$\mu_{0}$	-0.276	(<10^−3^)	-0.300	(<10^−3^)	-0.143	(<10^−3^)	0.023	(0.231)
$\sigma_{0}$	-0.011	(0.365)	0.079	(0.005)	-0.373	(<10^−3^)	-0.402	(<10^−3^)
*g*	0.593	(<10^−3^)	0.357	(<10^−3^)	-0.089	(0.004)	-0.038	(0.119)
ν	0.057	(0.032)	0.043	(0.083)	-0.009	(0.389)	0.030	(0.168)
*T*_*p*_	N/A		-0.622	(<10^−3^)	N/A		-0.076	(0.010)
**Parameter**	**Median[$N_{\text{f}}$] for 𝒩=10**				**Mean[$\mu_{\text{f}}-\mu_{0}$] for 𝒩=10**			
	**without weevil**		**with weevil**		**without weevil**		**with weevil**	
	**PRCC**	***p*-value**	**PRCC**	***p*-value**	**PRCC**	***p*-value**	**PRCC**	***p*-value**
$\mu_{0}$	-0.200	(0.310)	0.028	(0.147)	-0.482	(0.469)	0.420	(0.079)
$\sigma_{0}$	0.249	(0.220)	black!250.885	(0.051)	black!25-0.904	(<10^−3^)	black!25-0.878	(0.054)
*g*	black!250.932	(<10^−3^)	0.631	(0.169)	-0.432	(0.009)	-0.125	(0.373)
ν	-0.387	(0.190)	-0.725	(0.105)	-0.612	(0.079)	black!25-0.742	(0.075)
*T*_*p*_	N/A		-0.381	(0.291)	N/A		0.319	(0.153)

PRCC values indicating a strong correlation (i.e., |PRCC|>0.7) with a low *p* value (i.e., *p*<0.1) are indicated with gray shading.

**Fig 4:**
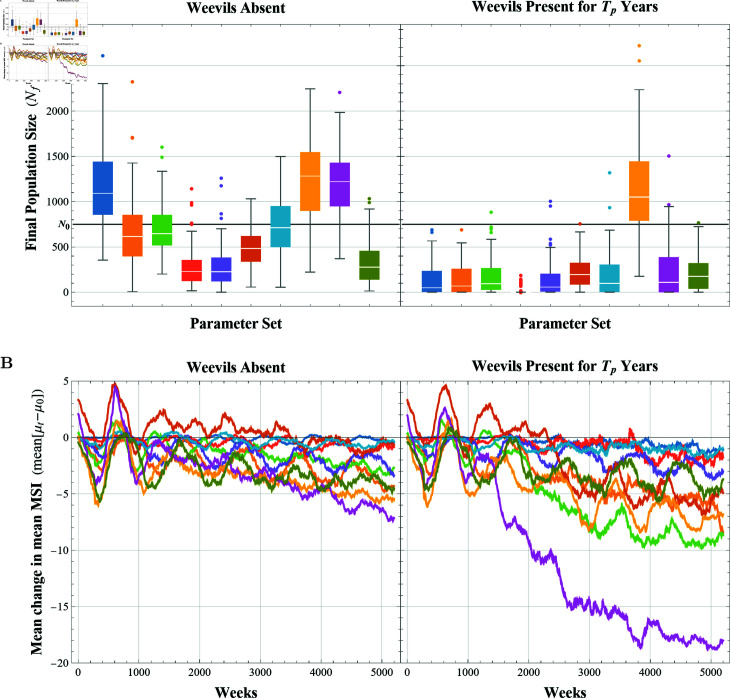
Distribution of final population size (A) and mean change in mean MSI over time (B) over 100 simulations for each parameter set shown in Table 3 in the absence of weevils (left) and with weevils present for *T*_*p*_ years (right); each color represents a single experiment (100 simulations of 1 parameter set). The bold line in (**A**) shows *N*_0_ = 750.

**Analysis with 𝒩=10 parameter sets.** Given the many elements of stochasticity within the model (see the Stochasticity section), it is possible to have significant variance in model outputs for a single parameter set. To explore the range in model output across replicate simulations of the same parameter sets, we generated 𝒩=10 parameter sets using LHS (see Table 3), and ran a single experiment (i.e., 100 replicate simulations) for each parameter set. All 10 experiments were simulated using the same initialized forest (see the Initialization section) and each simulation started with a population of 750 *T. utriculata* rosettes with an initial LLL ≥ 15 cm (i.e., $\ell_{i}(0)\geq 15$ cm). For each experiment, we calculated the mean final population size ($\text{mean}[N_{5200}]$) with and without weevil predation and the mean change in the MSI over time ($\text{mean}[\mu_{t}-\mu_{0}]$) with and without weevil predation. A graphical depiction of these results are given in Fig 4. Additionally, for each parameter set, we calculated the statistical significance of the difference of the mean $N_{\text{f}}$ with and without weevil predation and the mean $\mu_{\text{f}}-\mu_{0}$ with and without weevil predation (Student’s *t*-test with 95% confidence) the values of which are given in Table 3.

**Table 3:** Parameter sets generated through Latin hypercube sampling.

Parameter Set		$\mu_{0}$	$\sigma_{0}$	g	ν	$T_{p}$	p-value	
							$Mean[N_{\text{f}}]$	$Mean\left[\mu_{\text{f}}-\mu_{0}\right]$
1	∎	55.000	1.000	0.192	0.034	27.222	<10^−3^	0.144
2	∎	71.667	7.222	0.175	0.040	16.111	<10^−3^	<10^−3^
3	∎	48.333	8.778	0.158	0.026	21.667	<10^−3^	<10^−3^
4	∎	65.000	2.556	0.125	0.037	24.444	<10^−3^	0.290
5	∎	75.000	5.667	0.150	0.029	10.556	<10^−3^	0.692
6	∎	45.000	13.444	0.142	0.015	13.333	<10^−3^	<10^−3^
7	∎	61.667	4.111	0.167	0.018	18.889	<10^−3^	0.213
8	∎	58.333	11.889	0.200	0.021	5.000	0.057	0.149
9	∎	51.667	15.000	0.183	0.032	30.000	<10^−3^	<10^−3^
10	∎	68.333	10.333	0.133	0.023	7.778	<10^−3^	0.396

Varied parameters are the initial mean MSI ($\mu_{0}$), initial standard deviation in MSI distribution ($\sigma_{0}$), germination rate (*g*), MSI heritability variation (ν), and the length of the predation period (*T*_*p*_). Shaded *p*-values indicate a statistically significant difference in $\text{mean}[N_{\text{f}}]$ or $\text{mean}[\mu_{\text{f}}-\mu_{0}]$ between simulations with and without weevil predation.

The distribution of final population size at the final time ($N_{\text{f}}$) for each experiment (and thus, for each parameter set in Table 3) are shown in Fig 4A without weevil predation (left) and with weevil predation (right). In each graph, the horizontal line shows the initial population size *N*_0_ = 750. Without weevil predation, seven of the ten parameter sets lead to the median final population size below the initial (i.e., $\text{median}[N_{\text{f}}]<N_{0}$), though none of the parameter sets lead to a final population size distribution completely below the initial population size (i.e., $\text{max}[N_{\text{f}}]<N_{0}$). In contrast, with weevil predation, nine out of the 10 parameter sets lead to the median final population size below the initial population size, and three of the 10 parameter sets lead to the entire final population size distribution being below the initial population size. Upon examination of the statistical significance of the difference between $\text{mean}[N_{\text{f}}]$ with and without weevil predation, only parameter set 8 (see Table 3) showed no significant difference with a confidence level of 95%, though, parameter set 8 would have a significant difference with a confidence level of 90%. It should be noted that parameter set 8 happens to have the combination of greatest germination rate and the shortest period of weevil predation.

The mean change in mean MSI at each time step over 5,200 weeks for each experiment are shown in Fig 4B without weevil predation (left) and with weevil predation (right). Note that even in the absence of weevil predation, the mean MSI decreases over time (averaged across 100 simulations). However, in four out of the 10 parameter sets (shaded in Table 3), the mean change in the mean MSI at the final time without weevil predation is significantly higher than when weevil predation is present with a confidence level of 95%. This result does not change if the confidence level is changed to 90%.

Table 2 shows the PRCC values and corresponding *p*-values for each combination of parameter ($\mu_{0}$, *g*, ν, $\sigma_{0}$, and *T*_*p*_) and outcome ($\text{median}[N_{\text{f}}]$ without weevils, $\text{median}[N_{\text{f}}]$ with weevils, $\text{mean}[\mu_{\text{f}}-\mu_{0}]$ without weevils, and $\text{mean}[\mu_{\text{f}}-\mu_{0}]$ with weevils). PRCC values indicating a strong correlation (i.e., |PRCC|>0.7) with a low *p*-value (i.e., *p*<0.1) are indicated with gray shading in Table 2. The only PRCC value indicating a strong correlation without a *p*-value that met the 90% confidence threshold was the correlation between the variation of heritability in MSI (ν) and the median final population size with weevil predation. The significance of the PRCC results could be increased by increasing the total number of parameter sets; however, this would increase overall computational time.

## Discussion

The large, long-lived, epiphytic bromeliad *T. utriculata* has been subject to predation from the invasive weevil *M. callizona* since the late 1980s. These weevils lay their eggs in the tissue of bromeliads, resulting in the inability to reproduce. They preferentially select large bromeliads, leaving fewer large bromeliads to reproduce. As the size of induction is theorized to be a heritable trait, this could be causing the average size of induction in these populations to decrease over time. Many reproductive measures, such as seed fecundity, seed mortality, and germination rate, are tied to the size at induction. Thus, if the size at induction is decreasing, this may lead to a reduction in the fitness of the population over time, eventually causing extinction.

Analysis of simulations of our ABM show that in order for the population to survive for 100 years with weevil predation, the germination rate needs to be about 13–20%. This is considerably higher than the germination rates needed to survive 100 years without weevil predation, which falls around 10–12%. In order to have population growth rather than just survival, the germination rates need to be around 20%. Furthermore, PRCC results indicate that the germination rate is strongly correlated with the final median population size, meaning that germination rate may be one of the most important factors in maintaining these natural populations of *T. utriculata* in the absence of weevils. Populations with smaller mean sizes of induction have lower fecundity, increasing the importance of higher germination rates to maintain the population size. The germination rate of *T. utriculata* has not been rigorously studied. However, germination experiments on *T. utriculata* seeds harvested from Florida native *T. utriculata* have begun at The Kampong National of The Tropical Botanical Garden in Miami, FL. The results of these experiments will be integrated in future versions of this ABM.

Our model has also shown that there are conditions under which (given all model assumptions) the mean size of induction is, in fact, decreasing over time, confirming our initial hypothesis and field observations at MRSP. This has important ecological implications for *T. utriculata* populations because of the inherent ties between the mean induction size and population viability. A decrease in the MSI may imply a decrease in the populations’ ability to reproduce. Smaller bromeliads have lower mean seed fecundity. If this shift results in germination rates below 13% the populations will not be expected to survive even 100 years.

This could have implications on the community at large because bromeliads are known to provide habitats for many other species in the forest canopy. The overlapping leaves of the rosette form a tank that impounds water, forming unique phytotelm micro ecosystems housed within each tank bromeliad. Each rosette can contain dozens of invertebrate species, at least 15 of which are found only in the phytotelm of tank bromeliads native to Florida [10,18]. The *T. utriculata* rosettes are the among the largest of all native Florida tank-forming bromeliads and can hold up to a liter of water. They also have the widest range phytotelm invertebrates among the Florida large tank-forming bromeliads. Furthermore, larger bromeliads also have more diverse communities of bacteria with unique functional characteristics [19]. This means that *T. utriculata* in Florida provide the most opportunity to promote biodiversity through the formation of phytotelm and bacterial communities. The extirpation of this species would result in significant loss of biodiversity and ecosystem function across Florida and could have a significant impact on the ecological structure of the community in general.

Current conservation measures for *T. utriculata* include removing large *T. utriculata* rosettes from the forest, removing any weevils present, and placing the rosettes in mesh “conservation cages” that protect the rosettes from weevil infestations during induction, the building of the inflorescence, flowering, and dehiscing. Mature seeds from dehisced flowers are then propagated back into the canopy [15]. Experimental trials of this conservation method are underway in the Enchanted Forest Sanctuary in Brevard County, Florida and at Fakahatchee Strand Preserve State Park, Collier County, Florida. We plan to extend this model to examine the impact of such a conservation measure by modifying the submodels to account for the removal of large rosettes from the canopy and a protected reproduction period, followed by the targeted dispersal of the resulting seeds into the canopy.

Other future directions for this project include refining the model based on new vital rates data gathered for *T. utriculata* in Florida and explicitly modeling weevil movement rather than imply their presence in the calculation of vital rates. However, data on the movement patterns of ovipositing female *M. callizona* within the forest would be needed in order to accurately simulate the impact of the weevils upon the *T. utriculata* or other large bromeliad rosettes. Additionally, this model could be expanded to explore the potential long-term impact of conservation methods designed to remove the invasive weevil, minimize weevil damage of bromeliads, or repropagate populations. This model could also be adapted and expanded to simulate other large bromeliads native to Florida that are vulnerable to weevil predation, including *T. fasciculata* and *Guzmania monostachia*. In Florida forests where all three large bromeliad species are present, it is important to understand how weevil predation and potential conservation methods will impact individual bromeliad population and the entire bromeliad community with a forest.

In summary, we illustrate that the presence of the invasive *M. callizona* in Florida is likely to have an important and detrimental effect on local biodiversity if no conservation efforts are taken. The bromeliads in these populations are growing smaller, which will impede their ability to reproduce at a rate high enough to survive the presence of the weevils. The altered diversity and structure of communities will likely have far-reaching ecological consequences. We recommend conservation efforts to prevent significant loss of biodiversity.

## Methods and models

### Myakka River State Park (MRSP) data

Cooper [11] completed an extensive study of the populations of *T. utriculata* and *T. fasciculata* in MRSP that generated monthly data on the populations from June 2001 (about one year after *M. callizona* were first found in MRSP) to June 2005 that was used to quantify the impact of weevil predation. The monitored bromeliads were categorized into three size classes according to the LLL which approximately corresponds with the age of the rosette (Table 1). The transition from the small to medium size class corresponds to a morphological change in *T. utriculata* leaves. When a *T. utriculata* rosette reaches roughly 15 cm LLL, the leaf bases broaden which allows for the formation of a tank that can impound rain water [20]; Fig 1 shows this occurs around 5 years of age. Impounded rain water can be held for many months [21] providing water and nutrients for the rosette, and habitat for a variety of animals and plants [11,22]. No *T. utriculata* have been observed to have started building an inflorescence prior to the formation of their tank. Data from the Cooper study [11] was used to determine *T. utriculata* death rates from natural causes and due to weevil predation for the model presented in the Agent-based model description section. Additionally, the selection of field sites and monitoring protocol from the Cooper study [11] were used to inform further data collection described in the remainder of this section.

#### Data collection protocol.

To describe the size classes and spatial distributions of large, long-lived *Tillandsia* in a natural landscape with weevil impacts, we conducted an inventory within MRSP. Initial inventory work occurred between June 2001 and June 2005 [11]. At that time, while the weevil outbreak was accelerating, 10–100 bromeliads occurred within 13 distinct areas of the park, which were incorporated into a spatially stratified monitoring protocol. In each area, host plants were tallied and a subset were mapped and inventoried every six months. Inventory data included the number of apparent tillandsias in three size classes based on LLL (0–15 cm, 15–60 cm and > 60 cm). Among these plants, 739 were monitored monthly for survival, providing an estimate for mortality from weevils and other causes early during the infestation.

To obtain more high resolution data later during the weevil outbreak, in March 2018, we used standard forest inventory techniques to locate host trees [23], count tillandsias in different size classes, and map reproductive individuals within the canopy in one of the original 13 inventory areas (FPS research and collection permit no 11201714). Specifically, we obtained coordinates near the base of host trees (*Sabal palmetto*, *Pinus elliottii var. densa*, *Quercus virginiana*, and *Quercus laurifolia*) using a Trimble Juno 5B (Trimble Inc., Sunnyvale CA) and measured the distance and direction to the tree base using a 50 meter tape and compass respectively. We then measured the diameter of the tree above root flare using a diameter tape as well as the diameter of any independent stem that branched below 1.3 m height above the ground (diameter at breast height, DBH). We used trigonometric identities to calculate the center of the rooting position of the tree from the measured coordinates, distance, direction, and tree base diameter. After locating the host trees, we counted every individual of *T. utriculata* and *T. fasciculata* as determined by the shape of the plant, color of the leaves, and form of the reproductive structures when present. Based on the perceived longest leaf length of each individual, we assigned it to one of four size categories: 0–15 cm, 15–30 cm, 30–45 cm and > 45 cm; four categories were used instead of three because there were no long-lived tillandsias with LLL > 60 cm. For any individuals that were in bud, flower, fruit, or declining due to weevil damage, we collected additional location information. First, we used a 50 m tape and compass to measure the distance and direction (respectively) from the base of the tree to the base of the *Tillandsia*. Then, we used a clinometer to measure the angle of inclination from an observation point along the tape to the base of the plant. We calculated the 3 dimensional coordinates for each plant using trigonometric identities. The full data set is provided as supplemental information in S1 Data.

#### Summary of data.

#### Host tree data.

Forest inventory revealed that 18.6% of host trees were host to at least one *T. utriculata*, and of the host trees with *T. utriculata*, 81.8% had only a single *T. utriculata*. Additionally, 91.5% of host trees were host to only one *T. fasciculata*, and of the trees with *T. fasciculata*, 29.6% of trees were host to only one *T. fasciculata*.

#### Host tree density.

**Fig 5:**
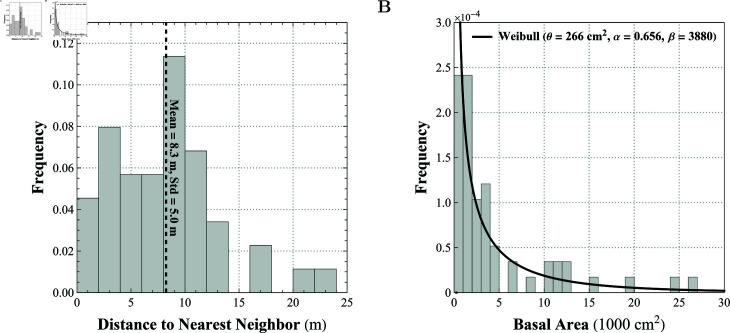
Histograms of (A) distance measured in meters to nearest neighboring tree given bin widths of 2 m (*n* = 44), and (B) basal areas of all sampled trees (*n* = 58; bin width $=1000\;{\text{cm}}^{2}$) with only fitted Weibull distribution shown, 𝒲(x;0.656,3880,266).

Using the geolocation (given in latitude/longitude coordinates) of each sampled tree (*n* = 44), the distance between all possible pairings of trees was calculated, and the distance to each tree’s nearest neighbor was calculated (mean  = 8.3 m; median  = 8.5 m; SD  = 5.0 m; min  = 0.9 m; max  = 23.8 m). A histogram of the distance to nearest neighbor with bin widths of 2 m is shown in Fig 5A.

#### Host tree basal area.

A histogram of the basal area of all sampled trees (*n* = 58) with bin widths of $1000\;{\text{cm}}^{2}$ is shown in Fig 5B. Sampled basal areas had range of $266\;{\text{cm}}^{2}$ to $70,845\;{\text{cm}}^{2}$. Parameter values for an exponential distribution, a gamma distribution, and a Weibull distribution were fitted using the maximum likelihood estimation technique in Mathematica 12.0. The estimated distribution parameters, Anderson-Darling statistic *p* value, and Akaike information criterion (AIC) for each distribution are shown in Table 4. Note that the exponential, gamma, and Weibull distributions have one, two, and three estimated parameters, respectively. The distribution that best represents the basal area data will have the highest *p* value for the Anderson-Darling statistic and the lowest relative value for the AIC. The distribution that fits these criteria is the Weibull distribution. The probability density function for the Weibull distribution can be expressed as

**Table 4:** Estimated parameters for distribution of basal area.

Distribution	Estimated Parameters			*p* value^*^	AIC
	Location	Shape	Scale		
Exponential	—	—	5685.05	0.00689	1120.88
Gamma	—	0.730507	7781.95	0.07150	1118.61
Weibull	265.901	0.655631	3879.71	0.47360	1095.25

^*^Anderson-Darling *p* value

$$𝒲(x;\alpha ,\beta ,\theta )=\left\{ \begin{array}{cc} (x-\theta {)}^{\alpha -1}\exp\left(-{\left(\frac{x-\theta }{\beta }\right)}^{\alpha }\right), & x>\theta \\ 0, & x\leq \theta \end{array}\right.,$$ (4)

where α, β, and θ are the shape, scale, and location (or threshold) parameters, respectively.

#### Demographic data for *T. utriculata* and *T. fasciculata.*

The demographic data for both *T. fasciculata* and *T. utriculata* collected in MRSP in March 2018 is shown in Table 5. Note that a total of 302 rosettes were documented (288 *T. fasciculata*, 14 *T. utriculata*). The Cooper study [11] documented a total of 296 rosettes in these same observations areas (70 *T. fasciculata*, 13 *T. utriculata*; 296 unknown), however, the observation period spanned two years. Distinguishing between the two species is challenging from a distance without an inflorescence. Standard characteristics used to distinguish these two species only develop in larger individuals, and the inflorescence in each species is distinctive.

**Table 5:** MRSP Data collected in March 2018.

Species	# Host Trees	Rosettes/Host Tree			# Rosettes with LLL (cm)				% Post- Induction
Combined	58	6.2	9.7	[1,58]	85	92	63	62	18.2%
*T. fasciculata*	54	5.9	9.2	[0,55]	84	90	58	56	18.4%
*T. utriculata*	11	0.3	0.8	[0,5]	1	2	5	6	14.3%

Rosettes that showed evidence of budding, flowering, fruiting, or post-flowering senescence were categorized as post-induction.

### Agent-based model description

We present a description of the model utilizing the Overview, Design concepts, and Details (ODD) protocol as described by Grimm, *et al* [24,25]. The ABM describe below has been programmed in NetLogo version 6.4, and is provided as supplemental information in S2 Model.

#### Overview.

This agent-based model is created to give a realistic look into the population dynamics of the *T. utriculata* with the presence of the invasive weevil *M. callizona*. The model includes growth and dispersal procedures informed by its life history and collected data. It also includes predation of the weevil population which can be set to occur for different lengths of time. Analysis of this model is intended to explain the impact weevil predation may have had on the timing of reproduction in *T. utriculata*.

#### Purpose.

The purpose of this model is to simulate populations of a Florida *T. utriculata* population in MRSP in the presence and absence of weevil predation. When simulated without weevil predation, model analysis can aid in the evaluation of the likelihood of recovery following weevil eradication. When simulated with weevil predation, model analysis can be used to examine population viability and shifts in reproductive timing of *T. utriculata*.

#### Agents.

There are two categories of agents in this model: (1) individual *T. utriculata* rosettes, and (2) the trees upon which the rosettes grow. Individual rosettes are not added as agents in the mode until they have reached 15 cm LLL (to conserve computational power); until that point, a count of all potential rosettes are stored in an array associated with mother rosette. The state variables for the ${\text{i}}^{\text{th}}$ *T. utriculata* rosette agent are given in Table 6. Demographic vital rates for rosette agents are primarily determined by the size of the rosette, measured as the LLL variable, $\ell_{i}(t)$ (see Table 6). The other rosette variable key to model analysis is *m*_*i*_, the minimum size for inflorescence induction (MSI), where the size is measured as LLL. The value of *m*_*i*_ is randomly sampled from a normal distribution whose mean is the MSI of the mother rosette and whose standard deviation is varied between simulations during model analysis (see Results section).

**Table 6:** Variables for the ${\text{i}}^{\text{th}}$ bromeliad agent representing a single *T. utriculata* rosette.

Variable	Type	Values	Description
(*x*,*y*)_*i*_	Integer	[0,173]×[0,173]	Patch location of the rosette
$\ell_{i}(t)$	Real	[15,105]	Length of longest leaf (cm)
*h*_*i*_	Integer	[2,15]	Height of rosette in canopy (m)
$B_{i}^{\text{alive}}$	Boolean	True/False	Status of having died (false) or not (true)
*a*_*i*_(*t*)	Integer	[0–max ticks]	Rosette age (wks)
$\gamma_{i}$	Integer	—	Rosette generation (from start of simulation)
$f(\ell_{i}(t))$	Integer	$0.026661\ell_{i}(t{)}^{2.376}$	# carpels on fully mature inflorescence
*s*_*i*_	Integer	—	Total # seeds produced during sexual reproduction
*m*_*i*_	Integer	[30,90]	MSI (cm)
*w*_*i*_(*t*)	Integer	*t* + 260	Time step at which offspring agents appear in model
${𝐎}_{i}$	Array	—	Offspring Array; each element is the number of seeds that survive to 15 cm LLL and become agents during a given week of the offspring emergence period
$B_{i}^{\text{induction}}$	Boolean	True/False	Status as pre-induction (false) or post-induction (true)
$\tau_{i}^{r}$	Integer	[71,111]	Time from induction to seed dispersal (wks); reproduction period
${\hat{\tau }}_{i}^{r}$	Integer	[0,$\tau_{i}^{r}$]	Counter to track reproduction period
$\tau_{i}^{s}$	Integer	[52,104]	Time from seed dispersal to senescence (wks); senescence period
${\hat{\tau }}_{i}^{s}$	Integer	[0,$\tau_{i}^{s}$]	Counter to track senescence period

The trees in the model, upon which the *T. utriculata* rosettes grow, are also agents. In the model, no distinction is made between the host tree species. The state variables of the ${\text{j}}^{\text{th}}$ tree agent are given in Table 7.

**Table 7:** Variables of trees located at coordinates (x,y).

Variable	Type	Values	Description
(*x*,*y*)_*j*_	Integer	[0,173]×[0,173]	Patch location of the tree
*b*_*j*_	Integer	~𝒲(x;0.655,3880,266)	Basal area (${\text{m}}^{2}$)
*c*_*j*_	Integer	0.0287*b*_*j*_ + 112.33	Crown area (${\text{m}}^{2}$)
$\eta_{j}$	Integer	[13,15]	Max height (m)
*d*_*j*_	Integer	[0.9,23.8]	Distance to nearest neighbor (m)

#### Patches and spatial scales.

The patches are arranged in a 173×173 grid, with each patch representing a $1\;{\text{m}}^{2}$ section of MRSP. In total, this area depicts approximately 3 hectares, the size of one inventory site [11]. If a patch has canopy cover and at least one height with a suitable branch, it is colored gray, otherwise the patch is colored black. Canopy patches each have a list of heights at which bromeliads may be located; the number of available heights are 0, 1, and 2 in the approximate ratio 60 : 36 : 4 (estimated based off field observations). Thus, 60% of patches with canopy coverage have no suitable branches to support a *T. utriculata*; 36% have branches at one height that can support *T. utriculata* rosettes; and only 4% have branches at two heights that can support *T. utriculata* rosettes. Canopy heights range from 2 m to the maximum height of the tree, and each tree is no taller than 15 m. The state variables of each patch are given in Table 8. The model has period boundary conditions with the landscape structured as a torus where the boundaries of the 173×173 grid wrap vertically and horizontally to simulate a continuous forest with no edge.

**Table 8:** Variables of patch located at coordinates (x,y).

Variable	Type	Values	Description
(*x*,*y*)	Integer	[0,173]×[0,173]	Location of the patch
pcolor	Color	black, gray	Color of that patch (grey = canopy coverage; black = no canopy)
${𝐡}_{\left(x,y\right)}$	Array	—	Array of canopy heights that can support rosettes; length ranges from 0 to 2 m
*L*_(*x*,*y*)_	Integer	[80,100]	Max leaf length (cm) available on patch at a single canopy height

#### Global parameters.

The model uses several global parameters to guide processes in the model. The parameters that are varied in the model analysis define the initial mean MSI ($\mu_{0}$), germination rate (*g*), MSI hertiability variation (ν), initial standard deviation in MSI distribution ($\sigma_{0}$), and the length of the predation period (*T*_*p*_). Additionally, there are several global parameters which are not varied, as well as global variables that are used to generate output for model analysis and model verification. The global parameters and variables are given in Table 9.

**Table 9:** Global parameters and variables for the model.

Parameter	Type	Values	Description
ticks	Integer	[0,5200]	Current time step (wks)
init-num-bromeliads	Integer	—	Initial number of bromeliads
germ-rate (*g*)	Real	[0.05,0.2]	Germination rate (%/wk)
MSI-hvar (ν)	Real	[0.015,0.04]	Heritability variation of MSI
init-mean-MSI ($\mu_{0}$)	Real	[45,75]	Mean MSI (cm) over initial bromeliad rosettes
init-std-MSI ($\sigma_{0}$)	Real	[1,15]	SD in MSI (cm) over initial bromeliad rosettes
predation-time (*T*_*p*_)	Integer	[5,30]	Years of weevil predation
weevil-begin	Integer	50−*T*_*p*_	Year at which weevil predation begins
weevil-end	Integer	50	Year at which weevil predation ends
total-available-space	Integer		Total ${\text{m}}^{3}$ available for bromeliads to grow
induction-LLL-list	Array	—	LLL (cm) at induction of all rosettes that reach induction
induction-age-list	Array	—	Age (in wks from germination) at time of induction of all rosettes that reach induction
MSI-list	Array	—	MSI (cm) of all rosettes that reach induction
MSI-generation-list	Array	—	Generation since initial population of all rosettes that reach induction
all-MSI-list	Array	—	MSI (cm) for every bromeliad agent in the model
init-MSI-list	Array	—	MSI (cm) for initial bromeliad population only
tp-converge	Boolean	0 or 1	Convergence reached for tree placement (0=no; 1=yes)

#### Time scales.

**Fig 6:**
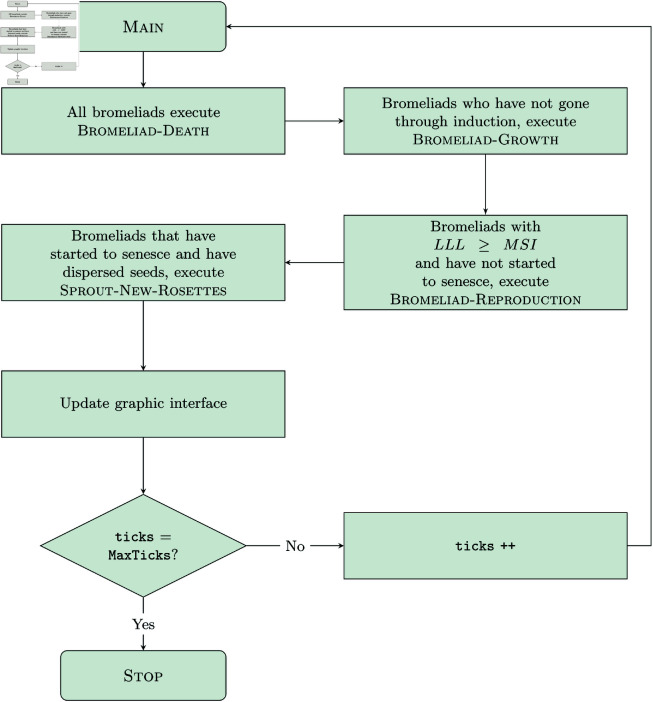
Flow diagram illustrating the sequence of procedures executed during one time step defined as tick, each of which represents one week. Green boxes indicate the execution of a submodel. See the Bubmodels section for descriptions of each submodel.

A single time step in the model represents one week and is tracked by the global variable ticks which is increased at the end of each iteration of the Main procedure (Fig 6). The simulation runs until ticks has reached the value of 5,200 weeks (i.e., 100 years).

#### Process overview.

The model is initialized with a mature forest and an established population of *T. utriculata*. The bromeliads are distributed throughout the forest according to probabilities generated from host tree data from MRSP (see the Myakka 457 River State Park (MRSP) data section). During each time step, there is some probability of each bromeliad dying from natural causes (overcrowding, falling off of a tree branch, predation, senescence, etc.). These probabilities vary according to the size of the rosette, and this process is encapsulated within the submodel Bromeliad-Death. Next, bromeliads that have yet to complete induction grow according to the Bromeliad-Growth submodel, which reflects the natural growth of each plant. Then, all bromeliads of reproductive size that have not yet dispersed seeds execute the Bromeliad-Reproduction submodel. In this submodel, rosettes complete inflorescence induction, generate the inflorescence, and store rosettes from resultant germinated seeds that survive to 15 cm LLL by calling the Seedling-Survival submodel. Next, post-reproductive bromeliads which are senescing execute the Sprout-New-Rosettes submodel. This submodel dictates the placement of the bromeliads that have grown to 15 cm LLL from seeds following a dispersal event. All submodels are described in the Submodels section. Finally, the graphic interface is altered to reflect changes in the agents’ sizes and life stage (vegetative, reproductive, senesced). Fig 6 illustrates the procedures that occur during each weekly time step.

#### Design concepts.

#### Basic principles.

The basic principles explored through this model are population viability and adaptation of reproductive timing in response to predation. There has only been one other study of population viability of *T. utriculata* in Florida [14], however that model did not allow for genetic drift or selection for certain values of MSI over multiple generations.

#### Emergence.

There are two system level properties that emerge from the model. The first is that over a 100 year period, the mean MSI decreases. This decrease is observed regardless of the parameter set and regardless of the presense of weevil predation. However, weevil predation and certain parameter sets allow for a greater decrease in the mean MSI over 100 years (see section on Exploration of the (*μ*_0_, *σ*_0_, *g*, *𝜈*, *T*_*p*_) parameter 484 space). The second property that emerges is a spatial clustering of rosettes within the model landscape.

#### Adaptation.

The MSI of each rosette is a heritable trait allowing for genetic drift and natural selection within the model. Thus, the distribution of MSI values within the population may adapt over generational time.

#### Sensing.

Bromeliads cannot sense their surroundings in this model; growth, reproduction, and death are determined based on the size or life-stage of the individual. Trees can sense their nearest neighbor, although this sensing is only used in the initialization of the model. Canopy covered patches can sense the LLLs of the bromeliads present on that patch. This is important in determining if a canopy patch is overcrowded.

#### Interaction.

There is competition for space among bromeliads located on the same patch at the same height. We assume that each height at every canopy patch can support a maximum total leaf length of *L*_(*x*,*y*)_. Larger bromeliads have two competative advantages: (1) their longer, broader leaves shade smaller rosettes, and (2) their larger tanks can impound greater amounts of water preventing them from drying out sooner. On patches with overcrowding, the smallest bromeliads on the patch will die (see submodel description Bromeliad-death). Beyond this, there is no bromeliad interaction. We assume pollination of flowers either through self-pollination or animal-mediated pollination. Trees and bromeliads interact only in that the bromeliads are epiphytic, living within the tree canopy.

#### Stochasticity.

Many processes within this model are dependent upon stochastic decisions. Whenever a set of agents is asked to execute a command or procedure, the order in which the individual agents complete the ask is randomized. We divide other stochastic agent processes into two categories.

#### Decisions using simple probabilities.

Each of the following are events that occur with probability *p*.

- During model initialization, each tree will host at least one bromeliad with probability *p* = 0.186. Of those trees, there is a probability *p* = 0.182 that the tree will host more than one bromeliad. Trees that host more than one bromeliad have an equal probability of hosting two, three, four, or five plants.
- During model initialization, a canopy patch will have 0, 1, or 2 available heights to host a bromeliad with probabilities *p* = 0.60, *p* = 0.36, and *p* = 0.04, respectively.
- During model initialization, for rosettes with an LLL greater than their MSI, there is a probability *p* = 0.66 that the individual is post-induction. Of those rosettes that are post-induction, there is a probability *p* = 0.5 that they are post seed-dispersal and undergoing senescence at *t* = 0.
- At each time step, bromeliads will die with a size-dependent probability as given in Table 1.
- Each bromeliad that has reached its MSI will begin reproduction with probability $p=1-0.05^{1/260}\approx 0.0115$ at each time step; calculated such that 95% of rosettes will go through induction within 5 years of reaching their MSI.

#### Decisions using probability distributions and weighted sets.

- During initialization, the basal area (*b*_*j*_) of each tree is generated by sampling a random number from the Weibull Distribution with parameters given in Table 4.
- During initialization, each tree is randomly assigned a maximum height ($\eta_{j}$) in meters sampled from the discrete uniform distribution 𝒰[13,15] based on MRSP Canopy Walkway height, average heights of *Sabal palmetto* [26]. For trees with available heights for hosting bromeliads, the heights are sampled from the uniform distribution $𝒰[2,\eta_{j}]$.
- During initialization, each patch with canopy coverage with a non-zero number of heights available, is randomly assigned a maximum total leaf length space available on the patch at a single canopy height (*L*_(*x*,*y*)_) from the discrete uniform distribution 𝒰[80,100].
- During initialization, the height of each bromeliad in the canopy is set randomly according to the available heights of the canopy patch on which the bromeliad has been placed.
- During initialization, each rosette’s initial LLL ($\ell_{i}(0)$) is sampled from an exponential distribution with mean 14.4 cm [11].
- During initialization, each rosette’s MSI (*m*_*i*_) is sampled from a truncated normal distribution over the range [30,90] cm, where the mean and standard deviation are varied in the model analysis (see the Results section).
- The time from induction to seed dispersal for each rosette ($\tau_{i}^{r}$) is sampled from a discrete uniform distribution 𝒰[71,111]; the time from seed dispersal to senescence for each rosette ($\tau_{i}^{s}$) is sampled from a discrete uniform distribution 𝒰[52,104] [8,11,21].
- The weekly growth rate of each bromeliad is sampled at each time step from a continuous uniform distribution to yield a ±5% deviation from the average growth rate using the size classes given in Table 1. The equation for weekly growth rate of each bromeliad is given in Eq (5) in the description of the submodel on Bromeliad-growth and depends on the time (in weeks) spent within a size class.
- The number of seeds each carpel produces for each bromeliad is determined from a normal distribution with a mean of 79.1 cm and standard deviation of 21.1 cm [16].
- The duration of each seed’s germination period is sampled from a gamma distribution with parameters α=5 and β=1 such that 99% germinate in 12 weeks [21,27].
- The location to which dispersed seeds travel is randomly selected from a weighted set of patches based on distance from the mother rosette. See description of the submodel on Sprout-new-Rosettes.

#### Collectives.

Collectives of patches are associated with a single tree agent to represent the canopy cover of that tree. These collectives are static over the course of a simulation.

#### Observations.

At initialization, the initial number of bromeliads and the initial mean and standard deviation of the MSI values are recorded. In addition, recorded forest measures include a histogram of the distance to the nearest neighboring tree, a histogram of the basal area of each tree, and the mean and standard deviations of the distance to the nearest neighboring tree.

As the model runs, the following measures are recorded once per year (i.e., recording every 52 time steps), the total bromeliad population of living rosettes above 15 cm LLL, the number of rosettes that have reached induction, the MSI values of all rosettes that have reached induction, and the mean and standard deviation of the MSI values of all rosettes that have reached induction.

#### Objectives, learning, prediction.

Neither the bromeliads nor trees have objectives, a learning capacity, or a predictive capacity in the model.

#### Initialization.

The environment is initialized in two steps: (1) Forest Creation, and (2) Initialization of Bromeliads. Each of these steps is written as a separate procedure so that they can be executed independently. This allows for multiple simulations using the same forest.

#### Forest creation.

In this procedure, the tree agents are created and initialized. Each tree is assigned a basal area (*b*_*j*_), crown area (*c*_*j*_), and a maximum tree height ($\eta_{j}$). From our data, the basal area of the trees in MRSP are randomly sampled from a Weibull distribution (see Eq (4)) with parameters α=0.656 (shape), β=3880 (scale), and θ=266 (location). Empirical research of southern live oak forests by Spector & Putz [28] provides an allometric relationship between crown area and basal area as $c_{j}=0.0287\;b_{j}+112.33$, where *b*_*j*_ is measured in cm^2^ and *c*_*j*_ is measure in ${\text{m}}^{2}$. The maximum tree height for each tree agent are drawn from a discrete uniform distribution over the interval [13,15] meters based on MRSP Canopy Walkway known heights.

Next, the trees are placed within the environment. Using data collected in MRSP, we calculated the density and distribution of host trees (see Myakka River State Park 593 (MRSP) data section). The data shows that the nearest neighboring host tree is on average approximately 8.3 m away with a standard deviation of approximately 5 m and a range of 0.9 m to 23.8 m. In the model, we mimic this distribution by first randomly placing 135 tree agents in the model landscape. Each tree then calculates *d*_*j*_, the distance to its nearest neighbor. Let ${\bar{x}}_{nn}$ be the sample mean of the nearest-neighbor distance and *s*_*nn*_ be the sample standard deviation of the nearest-neighbor distance. Next, the following sequence of operations is repeated until termination criteria are met.

- If *s*_*nn*_<4.0, then 5% of the tree agents are randomly selected and moved to random patches within the model landscape.
- Trees whose distance *d*_*j*_ is outside the data range are moved appropriately. For each tree,
  – if *d*_*j*_<0.9, then the tree agents is moved a distance of 0.9−*d*_*j*_ m away from its nearest neighbor, and
  – if *d*_*j*_>23.8, then the tree agent is moved a distance of *d*_*j*_−23.8 m closer to its nearest neighbor.
- Five percent of trees whose distance *d*_*j*_ is within the data range move slightly. For each tree that is moved,
  – if *d*_*j*_<8.3, then the tree agent moves 0.25 m in the direction away from its nearest neighbor ±5 degrees, and
  – if *d*_*j*_>8.3, then the tree agent moves 1 m in the direction closer towards its nearest neighbor ±5 degrees.
- For each tree *d*_*j*_ is recalculated.
- The values ${\bar{x}}_{nn}$ and *s*_*nn*_ are recalculated.

The termination criteria are (1) once the sequence has repeated 50,000 times or (2) when $|{\bar{x}}_{nn}\;-\;8.3|\leq 0.5$ and $|s_{nn}\;-\;5.0|\leq 0.5$. Forests with 125 trees (over $29,929\;{\text{m}}^{2}$) converged, on average, in 11,174 iterations. Forests with fewer than 105 trees or more than 140 trees will not reliably converge with this method.

The patches within the crown area of each tree are then assigned a number of heights that are available to host bromeliads. Canopy patches have zero, one, or two available heights in the approximate ratio 60:36:4. The specific heights are assigned randomly between 2 m and the maximum height of the tree. Patches with one or two available heights are colored gray. Lastly, each tree is assigned a variable indicating the number of bromeliads with which it will be initialized. Our 2018 MRSP *T. utriculata* data showed that 18.6% of trees in the forest were host to at least one *T. utriculata*, and of the trees with bromeliads, 81.8% have a single *T. utriculata*. The remaining have an equal probability of hosting two, three, four, or five *T. utriculata*.

#### Initialization of bromeliads.

In this procedure, each tree is populated with an initial number of bromeliads (all *T. utriculata*) and the variables of each created bromeliad agent are initialized. Tree by tree, the preassigned number of bromeliads are generated and placed randomly within the canopy; height is selected randomly from the available heights at that patch. To initialize a forest with a *T. utriculata* population similar to what was found prior to the population decline from weevil predation, each tree receives 10 times the number of bromeliads as would be indicated by our 2018 MRSP data to represent pre-predation population sizes. Each bromeliad is assigned an initial LLL ($\ell_{i}(0)$) from an exponential distribution with mean 14.37 cm, and an MSI (*m*_*i*_) from a truncated normal distribution with mean $\mu_{0}$, standard deviation $\sigma_{0}$, and range [30,90] cm. Of the bromeliads with $\ell_{i}(0)>m_{i}$, one-third are pre-induction (i.e., $B_{i}^{\text{induction}}=\text{false}$), one-third are in the reproduction period (i.e., $B_{i}^{\text{induction}}=\text{false}$ and ${\hat{\tau }}_{i}^{r}>0$), and the remaining one-third are in the senescence period (i.e., $B_{i}^{\text{induction}}=\text{false}$ and ${\hat{\tau }}_{i}^{s}>0$).

#### Submodels.

The submodels within the ABM are described in detail in this section. Within ODD protocols, submodels are also called *procedures*.

#### Bromeliad-death.

***Procedure executed by the main procedure.*** For *T. utriculata* rosettes, death can occur at each time step (i.e. each week) by natural causes or from weevil predation. Natural death occurs when a rosette is knocked to the ground from natural weather events (denoted as natural death, non-crowding), when overcrowding leads to lack of resources and eventually death (denoted as natural death, crowding), or after a rosette has dispersed its seeds and the rosette naturally desiccates over a lengthy senescence period, and eventually dies (denoted as senescence death). The order in which we consider the possibility of each type of death occurring to rosettes within the population follow the order in which they are described below. For each type of death, if a rosette dies, its Boolean variable $B_{i}^{\text{alive}}$ will be set to false. The rosette agent is only removed from the simulation if it has no seeds left to disperse.

**Fig 7:**
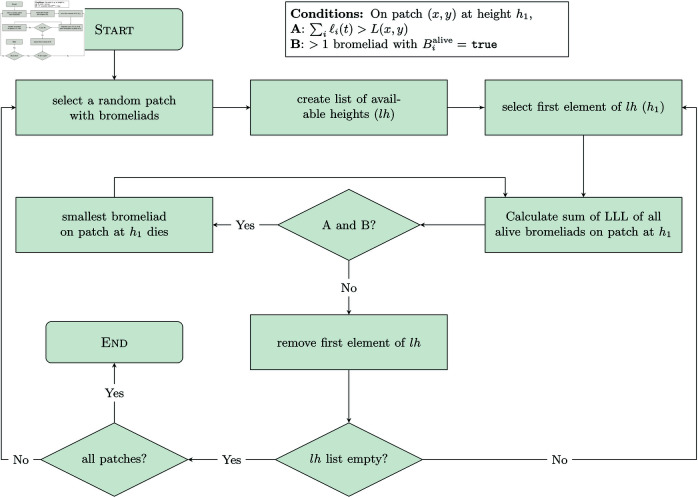
Flow diagram representing bromeliad death by overcrowding within the Bromeliad-death procedure.

- **Natural death, non-crowding.** The weekly probability that a *T. utriculata* rosette of a particular size class will die from natural death, non-crowding was calculated based on data collected in MRSP [11]. There is a 0.976% and 0.400% weekly probability of natural death, non-crowding for rosettes of size LLL = 15–30 cm and of size LLL > 50 cm, respectively. If a rosette dies, its Boolean variable $B_{i}^{\text{alive}}$ is set to false.
- **Senescence death.** After an individual releases seeds, the rosette begins to senesce and will die within the next year or two [8,21]. In the model, the duration of the senescence period ($\tau_{i}^{s}$) for each individual is determined at the time of seed dispersal (see description of submodel for Bromeliad-reproduction). A counter (${\hat{\tau }}_{i}^{s}$) is used to count up to the full length of the senescence period. If ${\hat{\tau }}_{i}^{s}>\tau_{i}^{s}$, then the rosette’s Boolean variable $B_{i}^{\text{alive}}$ is set to false, otherwise the counter ${\hat{\tau }}_{i}^{s}$ is increased by 1.
- **Natural death, crowding.** Bromeliads are also susceptible to dying when there is local overcrowding. A patch at location (*x*,*y*) has an array variable called ${𝐡}_{\left(x,y\right)}$ which contains a list of all canopy heights that can support rosettes, and a variable *L*_(*x*,*y*)_ which denotes the maximum total leaf length space available at each canopy height. Each patch has either 0, 1, or 2 available heights. Fig 7 shows the process for how natural death by overcrowding occurs.
  Each patch (*x*,*y*) with any bromeliads at that location, creates a temporary list of all available heights (denoted *lh*). The first element of the list (denoted *h*_1_) is selected. On patch (*x*,*y*), at the height *h*_1_, the sum of the LLL for all living bromeliads is calculated (denoted $S_{\left(x,y,h_{1}\right)}$). If $S_{\left(x,y,h_{1}\right)}>L_{\left(x,y\right)}$ and there is more than one bromeliad alive on patch (*x*,*y*) at height *h*_1_, then the $B_{i}^{\text{alive}}$ variable of the smallest bromeliad on that patch at height *h*_1_ is set to false. This process is repeated until either $S_{\left(x,y,h_{1}\right)}\leq L_{\left(x,y\right)}$ or there is only one bromeliad remaining on patch (*x*,*y*) at height *h*_1_. Then, the first element of *lh* is removed. If the list is non-empty, the new first element of the list is set to *h*_1_ and the process is repeated. If the list is now empty, then the procedure moves on to the next patch containing live bromeliads until all patches containing live bromeliads have been selected.
- **Death by Weevil predation.** The weevils have a demonstrated preference for larger rosettes [13], and thus when weevil predation is present demographic structure dictates the impact of weevil predation. Let *N*_*L*_(*t*) be the number of bromeliads with $\ell_{i}(t)\geq 50$, and *N*_*M*_(*t*) be the number of bromeliads with $30\leq \ell_{i}(t)<50$. If weevil predation is occurring in the model at the given time step, then all live bromeliads (i.e., with $B_{i}^{\text{alive}}=$ true) die from weevil predation with the following probabilities (Table 1):
  – 0.631% if $\ell_{i}(t)>50$ and *N*_*L*_(*t*)>0
  – 0.708% if $30\leq \ell_{i}(t)<50$ and $N_{L}\leq 10$
  – 0.708% if $15\leq \ell_{i}(t)<30$ and *N*_*L*_ = 0 and $N_{M}\leq 50$.
  Essentially, the largest rosettes are impacted by weevil predation first and smaller rosettes are not impacted by weevil predation when there are a sufficient number of larger rosettes available.
- **Removal.** Once all the forms of bromeliad death have been executed, dead individuals (i.e., $B_{i}^{\text{alive}}=$ false) with no seedlings with LLL less than 15 cm left (i.e., all values of ${𝐎}_{i}$ are 0) will be removed from the model.

#### Bromeliad-growth.

***Procedure executed by pre-induction bromeliad agents.*** Pre-induction bromeliads grow at a rate that depends on the size class of the individual. The average weekly growth rates of *T. utriculata* are based the size classes and corresponding ages given by [15] and are included in Table 1. In the model, only bromeliads with longest leaf length greater than 15 cm (i.e., in the medium and large size classes) are explicitly models as agents. The longest leaf length ℓ of bromeliad agent *i* is increased from time step *t* to time step *t* + 1 by

$$\ell_{i}(t+1)=\ell_{i}(t)+\alpha \cdot \frac{\text{avg }\Delta \text{ in LLL of size class (in cm)}}{\text{time in size class (in wks)}},$$ (5)

where α is randomly sampled from a uniform distribution over the interval [0.95,1.05] to simulate a ±5% variation in the growth rate at each time step, and the growth rate (avg Δ in LLL of size class (in cm)/time in size class (in wks)) is $\frac{8}{52}\;\frac{\text{cm}}{\text{wk}}$ for rosettes with LLL ∈[50,90] and $\frac{5}{52}\;\frac{\text{cm}}{\text{wk}}$ for rosettes otherwise.

#### Bromeliad-reproduction.

***Procedure executed by bromeliad agents that have reached their MSI.*** Reproduction in this model, refers to the processes leading to seed dispersal, occurring in the period of time after induction (see Fig 1). These processes include induction (the start of the production of the inflorescence), the growth of the inflorescence (including flower production and seed maturation), and the completion of sexual reproduction (i.e., when seeds are ready to disperse). Within this submodel, a bromeliad agent will update as follows (based on where it is in the process of reproduction):

- **Sexual reproduction complete.** If the reproduction period is complete (i.e. ${\hat{\tau }}_{i}^{r}>\tau_{i}^{r}$), then the time at which bromeliad *i*’s offspring will appear as agents within the model is set to *w*_*i*_(*t*) = *t* + 260, where *t* is the current time step. Next, the Seedling-Survival procedure is called. Lastly, the value of the senescence period $\tau_{i}^{s}$ is randomly sampled as an integer from a uniform distribution over the interval [52,104] weeks [8,11,21].
- **Growth of inflorescence, flowering, and seed maturation.** If the rosette is post-induction (i.e. $B_{i}^{\text{post-induction}}=$ true), then ${\hat{\tau }}_{i}^{r}$ is incremented by 1.
- **Start production of inflorescence.** If the rosette is pre-induction (i.e. $B_{i}^{\text{post-induction}}=$ false), then with probability 1.15% the rosette enters the post-induction phase (the probability is calculated such that, on average, 95% of all rosettes go through induction within five years of reaching their MSI). Upon entering the post-induction phase, the reproduction period $\tau_{i}^{r}$ is set to a value randomly sampled from a uniform distribution over the interval [71,111] weeks [8,11,21], and the value of $B_{i}^{\text{post-induction}}$ is set to true.

#### Seedling-survival.

***Procedure executed by bromeliad agents as they enter the senescence period.*** Seed germination occurs soon after seed dispersal, typically within a few months. However, in the model, bromeliad offspring rosettes are not added as agents until they have reached 15 cm LLL to conserve computational power. On average, growth from germination up to the 15 cm LLL takes five years [11,15,21].

The number of carpels on the inflorescence is related to the LLL by the function


$$f(\ell_{i}(t))=0.026661\ell_{i}(t{)}^{2.376},$$

where $\ell_{i}(t)$ is the LLL of rosette *i* at time step *t* [14]. The number of seeds in each carpel is then determined by a normal distribution with a mean of 79.1 seeds per carpel and a standard deviation of 21.1 [16]. Of the seeds generated, only a small percentage will germinate [11], however the exact germination rate is unknown and thus varied in the model analysis as the global parameter *g*. Of the germinated rosettes, only a small portion survive five years to reach 15 cm LLL (about 1.89% when no weevil predation is present; about 1.95% when weevil predation is present) [11]. These surviving offspring are stored as integers in the array ${𝐎}_{i}$ of the parent bromeliad such that they are added to the model as agents at the appropriate time (i.e. weeks to germination + 5 years to grow to 15 cm LLL; see Sprout-new-Rosettes procedure for details). Every new bromeliad that will survive to the small size class is assigned a number of weeks until germination based on a gamma distribution with a shape of 5 (α=5) and a scale of 1 (β=1), such that 99% of the seeds will germinate within 12 weeks [21,27].

#### Sprout-new-Rosettes.

***Procedure executed by bromeliad agents 260 – 286 wks past seed dispersal*** In Fig 1, seed dispersal occurs at the completion of building the inflorescence, flowering, and maturation of seeds. We will refer to the reproducing rosette as the “mother rosette”. The offspring of a mother rosette will enter the model as agents 260 – 286 wks after the seed dispersal of the mother rosette. We will refer to this 26-week window as the “offspring emergence period.” Note, spatial distribution of offspring rosettes within the model occurs at the time they enter the model as agents, not at the time of seed dispersal.

Mother rosette *i* has an array (${𝐎}_{i}$) that is defined during the Seedling-survival procedure, where each element $(o_{i}{)}_{j}$ of the array represents the number of germinated offspring that survive to 15 cm LLL and become agents during week *j* of the offspring emergence period. For mother rosette *i* on week *j*, $(o_{i}{)}_{j}$ offspring rosettes are dispersed into the canopy using a weighted dispersal function, which depends on the height in the canopy *h*_*i*_ of the mother rosette. Offspring rosettes can disperse to patches within a radius of dispersal $\frac{1.32}{0.65}h_{i}$ (based off wind tunnel experiments by [16]) that have canopy coverage and available heights within the range *[*2,*h*_*i*_ + 1*]* m above ground. Within that radius, the patch the offspring disperses to is determined randomly and is weighted by $\left((\text{distance to mother rosette }i)+1\right){\;}^{-1/2}.$ If the patch the offspring rosette disperses to has more than one available height, the height of the offspring is randomly selected among the available heights.

After the rosette has dispersed, its agent variables are initialized. Of note, its age is set to 260 weeks, and its MSI is randomly sampled from a normal distribution with a mean equal to the mother rosette’s MSI (*m*_*i*_) and a standard deviation equal to $\nu \times m_{i}$ [29].

## Supporting information

S1 Data MRSP Tree Host Data. CSV file of the host tree data taken in Myakka River State Park (MRSP).
(CSV)

S2 Model ABM NetLogo File. Netlogo file of the agent-based model described in the Agent-based model description section. Requires NetLogo version 6.4 or higher to run (https://ccl.northwestern.edu/netlogo/download.shtml).
(ZIP)
